# Improvement of automated analysis of coronary Doppler echocardiograms

**DOI:** 10.1038/s41598-022-11402-6

**Published:** 2022-05-06

**Authors:** Jamie Bossenbroek, Yukie Ueyama, Patricia E. McCallinhart, Christopher W. Bartlett, William C. Ray, Aaron J. Trask

**Affiliations:** 1 Department of Computer Science and Engineering, The Ohio State University College of Engineering, Columbus, OH USA; 2 Battelle Center for Mathematical Medicine, Columbus, OH USA; 3 Center for Cardiovascular Research and The Heart Center, The Abigail Wexner Research Institute at Nationwide Children’s Hospital, Columbus, OH USA; 4 Department of Pediatrics, The Ohio State University College of Medicine, Columbus, OH USA

**Keywords:** Computational biology and bioinformatics, Physiology, Cardiovascular biology

## Abstract

Coronary artery disease is the leading cause of heart disease, and while it can be assessed through transthoracic Doppler echocardiography (TTDE) by observing changes in coronary flow, manual analysis of TTDE is time consuming and subject to bias. In a previous study, a program was created to automatically analyze coronary flow patterns by parsing Doppler videos into a single continuous image, binarizing and separating the image into cardiac cycles, and extracting data values from each of these cycles. The program significantly reduced variability and time to complete TTDE analysis, but some obstacles such as interfering noise and varying video sizes left room to increase the program’s accuracy. The goal of this current study was to refine the existing automation algorithm and heuristics by (1) moving the program to a Python environment, (2) increasing the program’s ability to handle challenging cases and video variations, and (3) removing unrepresentative cardiac cycles from the final data set. With this improved analysis, examiners can use the automatic program to easily and accurately identify the early signs of serious heart diseases.

## Introduction

Coronary microvascular disease (CMD) is a heart condition affecting the smaller blood vessels that branch off from the main coronary arteries. Impairments in the coronary microcirculation disrupt the healthy regulation of myocardial blood flow and nutrient exchange^1,2^. CMD is a nonobstructive coronary artery disease, meaning that although there is no physical blockage, oxygenated blood is unable to move through smaller blood vessels at an adequate rate to maintain physiological demand^3^. This condition has been shown to be strongly associated with diabetes, and when paired with myocardial ischemia and myocardial diseases it is referred to as nonobstructive coronary artery disease (INOCA). CMD is one of the earliest signs of heart disease which can lead to myocardial infarction, heart failure, and/or stroke^1,2^. Functional, structural, and biomechanical deficits in coronary resistance microvessels (CRMs) are associated with CMD and are indicators that can be observed before the appearance of symptoms such as atherosclerosis (blockages in the arteries)^2^. With early and accurate identification of CMD, more serious and life-threatening cardiac conditions can be treated and prevented before they become deleterious.

While indirect methods to diagnose CMD are available, they are fraught with subjectivity. Positron emission tomography (PET) and magnetic resonance imaging (MRI) offer value in identifying impairments in cardiac perfusions, but currently include no direct measures to diagnose CMD^4^. Transthoracic Doppler echocardiography (TTDE) is an affordable and non-invasive method used to assess cardiovascular function through direct measurements of coronary blood flow (CBF), with potential to assess CMD. CBF is measured from one of the main coronary arteries under both baseline and stress (hyperemic) conditions, and this yields uniquely characteristic flow patterns in which diastole predominates and which can be analyzed to indicate impaired CBF^5^. For example, coronary flow velocity reserve (CFVR) is indicative of the amount of additional blood flow that the microvasculature can carry under stress; CFVR is lower in cases of coronary artery disease, even in otherwise asymptomatic subjects or in patients with INOCA^6^. This change in CBF likely represents a combination of functional, structural, and biomechanical impairments^1^. For example, previous studies by our laboratory observed inward hypertrophic remodeling associated with reduced CBF. This structural remodeling occurred before occlusive macrovascular atherosclerosis, which emphasizes the importance of early examination of coronary microcirculation^7^. However, manual analysis of TTDE CBF can be time consuming and subject to both intra-rater and inter-rater bias^8^. To resolve these issues, our groups began developing a MATLAB program to automatically extract data values from coronary flow patterns of TTDE video files^9^.

For each cardiac cycle in the TTDE flow pattern, the original MATLAB program automatically extracted several parameters including the peak velocity and velocity time integral, which are commonly used to quantify coronary health. CFVR was then calculated as the average peak hyperemic velocity divided by the average peak baseline velocity. When analyzing 98 baseline files and 117 hyperemic files both manually and with the automatic program, linear regression analysis showed significantly reduced variability when using automatic analysis, and the time to analyze videos was reduced from 1500 to 50 min. However, agreement between manual and automatic parameter output ranged from less than 1% difference to over 55% difference for certain variables^9^. While the accuracy of the simple regression model is comparable to human evaluators, the parameter variability suggested that with continued testing and program adjustments, automatic analysis of TTDEs could become increasingly more accurate and capable of processing challenging videos.

Extensive testing identified several potential areas in which improved analysis was possible, including the removal of interfering noise, the identification and analysis of fainter cardiac cycles, and the verification of peak selection in the ECG region. The original program was also limited to a single video height and width in pixels, which excluded the analysis of many Doppler videos. In this study, we present the results of an effort to improve the accuracy of the first-generation program through development of several key areas of analysis. The original program was developed in MATLAB but was recapitulated in Python in order to leverage OpenCV for computer vision and Google’s TensorFlow for downstream machine learning. Therefore, we have implemented an updated and improved program for extracting cardiac Doppler parameters from the Doppler videos using Python and a variety of best-in-class open-source Python libraries for image and signal processing. This approach allows easier distribution and community maintenance of the software, and it enabled us to address several of the data-processing limitations inherent in the MATLAB version as well. As Python is also an industry standard for machine learning development, changing to that environment allows us to leverage innovations in machine learning from both academia and industry much faster going forward.

**Figure 1:**
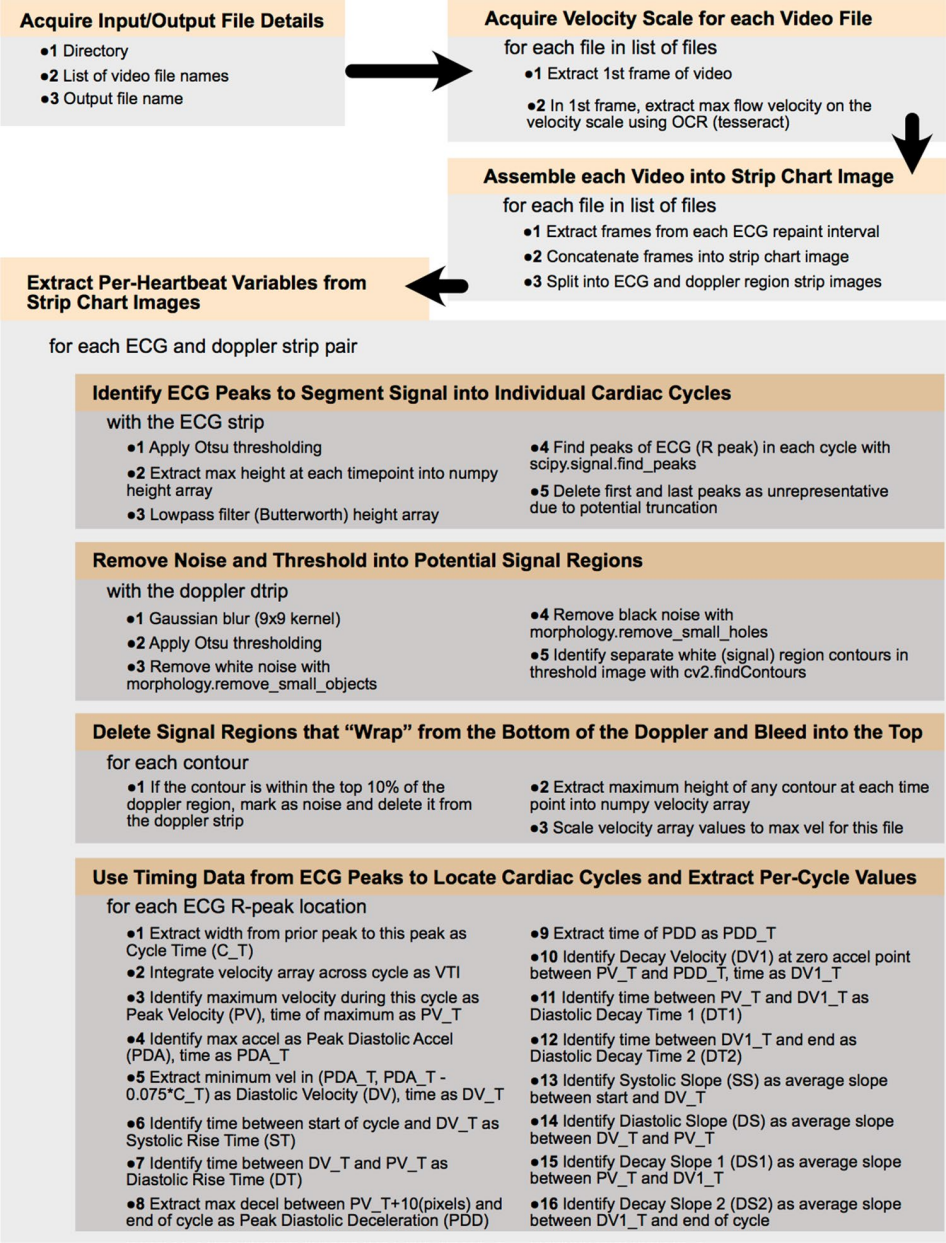
A diagram of the conceptual blocks and logic flow of the new Python version of the software.

We hypothesized that the use of OpenCV and modified heuristics could better address the original program’s limitations, and that these refinements would produce a comprehensive and accurate method for examiners to classify coronary flow issues through interpretation of CFVR values and other patterns in parameter output. These improvements allow examiners to take advantage of the speed and consistency offered by automated analysis without sacrificing diagnostic accuracy in assessing coronary diseases. A diagram of the conceptual blocks and logic flow of the new Python version of the software is shown in Fig. 1.

## Materials and methods

TTDE video files with approximately 20 distinct heart beats each were acquired from 12-week, 16-week, and 36-week old normal Db/db and type 2 diabetic (T2DM) db/db mice (Jackson Laboratories) at both baseline and hyperemic (high flow) conditions^10^. Doppler readings were measured at 1% isoflurane (baseline) and 3% isoflurane (hyperemia), and all measurements were taken from the left main coronary artery of the mice as previously described^10^. These videos were exported as .avi files from the VevoLab 3.1.1 software and analyzed offline using the improved program. Mice were housed under a 12-h light/dark cycle at 22 °C and 60% humidity. They were allowed ad libitum access to water and were fed standard laboratory mice chow. This study was conducted in accordance with National Institutes of Health Guidelines and was approved by the Institutional Animal Care and Use Committee at the Abigail Wexner Research Institute at Nationwide Children’s Hospital.

### Algorithm description

The improved program was written in Python, and utilized the following libraries: sys, cv2 (OpenCV), PIL, scipy, skimage, matplotlib, tkinter (Tk), pandas, and numpy. Initial data processing began with prompts to select the folder containing the video files to be analyzed, input a name for the output excel file, select the type of analysis as ‘Doppler’ or ‘Combined’ (the latter including analysis of color mode videos), and finally to select each video file to be analyzed. The new interface expanded on the functionality of the original program by allowing more than one baseline and/or hyperemic video file to be selected for analysis in each run as well as by accepting videos with any pixel height and width. The user was then prompted to enter the peak velocity value in mm/s on the Doppler window’s scale for each video, as well as the probe angle and minimum/maximum penetration in mm from the B Mode window if combined analysis was selected.

**Figure 2:**
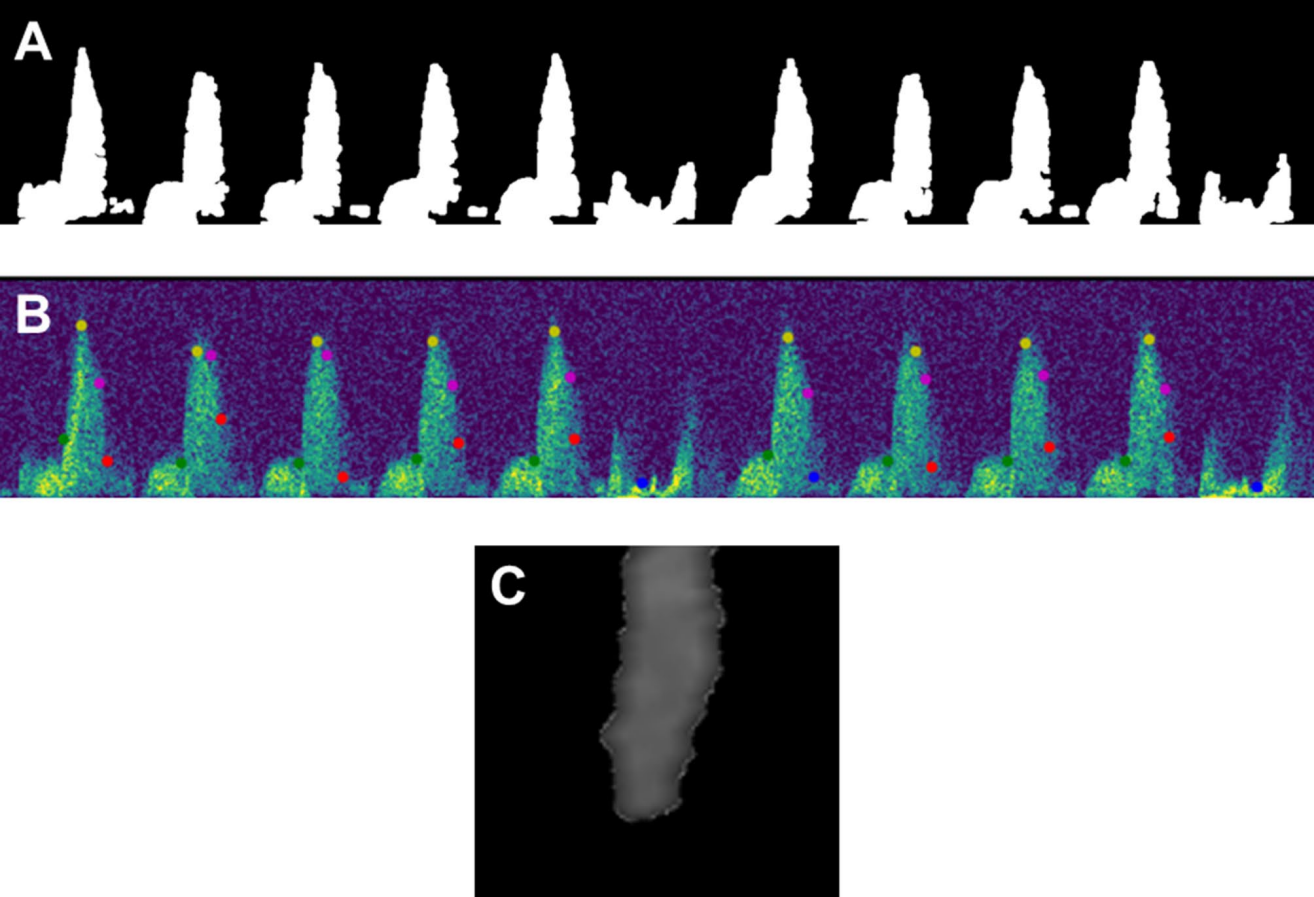
Images representing the steps taken by the Python algorithm for Doppler video analysis. Panel (**A**) displays a binarized image of the Doppler region, panel (**B**) shows the generated output image with critical values labeled with colored points, and panel (**C**) is an example of a vessel diameter measured during Color Mode analysis.

Once all parameters had been entered, the program parsed each video by inspecting the difference between subsequent video frames to identify frames where the scroll bar reset from the right to the left side of the Doppler window. These frames were concatenated into a single continuous image which was then cropped to the region of interest containing the coronary flow pattern and electrocardiogram (ECG) recording. A Gaussian filter was applied, the image was dilated with a linear structuring element, and then a global threshold value was calculated using OpenCV and Otsu’s method for image binarization. A representative binarized image is depicted in Fig. 2A.

The calculated threshold value was adjusted by the user in a ‘Scroll Test’ window, where the threshold was incremented or decremented to visually inspect how the level of filtering would affect the amount of data captured in the binarized Doppler image. Increasing the threshold value removed additional noise, while decreasing the threshold expanded the included envelope. Once the threshold value was verified, the program removed any remaining noise and filled small holes in the image using OpenCV. The updated program applied more complete noise removal by identifying all contours in the image and removing any objects that were not within ten pixels of the horizontal baseline. This corrected for any small noise objects and was especially useful in removing any ‘top noise’ located at the top of the Doppler window. Next, the program split the Doppler region into cardiac cycles by identifying peaks in the corresponding ECG pattern. Peaks were initially identified using scipy, and then an added check compared the distance between each peak to remove extra peaks that were too close together and to fill larger empty gaps with estimated peak locations. Finally, the program extracted the following parameters from each cardiac cycle: peak velocity, diastolic velocity, decay velocity, systolic rise time, diastolic rise time, diastolic decay time, systolic slope, diastolic slope, decay slope, heart rate, and velocity time integral. These were the same parameters extracted in the original MATLAB program^9^. All parameters were output to a Microsoft Excel file in .xlsx format.

The program generated an image of the coronary flow pattern with diastolic velocity (indicating the beginning of the diastolic phase), peak velocity (maximum velocity for each cycle), decay velocity (point at which acceleration switches signs closest to peak diastolic deceleration), peak diastolic deceleration (minimum acceleration), and end of cycle (correlating with the peaks found in the ECG region) indicated with green, yellow, pink, red, and blue points, respectively. An example of this image is depicted in Fig. 2B. The plots and corresponding output parameters generated by both the MATLAB and Python programs were inspected to find discrepancies where algorithmic or heuristic improvements might increase analysis accuracy.

The program also included an option for analysis of coronary diameters B-mode color videos, which is required to calculate CBF^10^. The algorithm began by masking the first frames of both the color mode and corresponding Doppler videos and identifying the borders of the B mode window and the center lines indicating where vessel measurements were taken from. For each video frame of the color mode file, these values were used to crop to a region around the center location before rotating the image based on the angle of the probe. The corresponding length of each pixel in mm was calibrated from the minimum and maximum probe depths entered by the user at the beginning of video analysis. The program then masked the image and identified any contours, and if the contour was large enough and in the correct location to exclude noise or ventricle filling, the diameter of the identified vessel was then calculated by finding the average distance between the left and right vessel walls of the object. The program output the minimum, maximum, mean, median, mode, and standard deviation of all diameters for each analyzed video. An example of a measured vessel is shown in Fig. 2C.

### Methodology

A collection of 18 Doppler video sets evenly distributed between 12-, 16-, and 36- week old healthy and diabetic mice were processed with both the original MATLAB program and the improved Python program. All tests were performed on the same computer by a single tester who entered in any prompted values and adjusted the threshold value for binarization as needed to fully capture the coronary flow pattern without including noise. Each video set included one baseline and one hyperemic video, and the Python program also analyzed the corresponding color mode videos acquired at baseline and hyperemic conditions. Videos were intentionally selected by the tester through visual inspection of video files in order to demonstrate a wide range of processing difficulty, from videos containing distinct Doppler regions with little noise to videos that the MATLAB program struggled to handle. Some challenging patterns included interfering noise or ‘top noise’ descending from the top of the Doppler image, poor contrast between background noise and the Doppler signal, and inconsistent ECG readings that led to the incorrect separation of cardiac cycles. Testing with the improved program could then demonstrate through specific examples that modified heuristics were better able to handle challenging videos, while videos with clearer signals that had already been fully captured by the original program continued to generate similar data.

### Statistics

The table of parameters for each cardiac cycle generated by the two programs were saved to a Microsoft Excel file, and for each parameter the mean and standard deviation (SD) across all cycles were calculated. With color mode analysis included, CBF could be calculated using the equation as previously described by us^10^:

$$CBF\left( {\text{mL/min}} \right) = \left( {\left( {\uppi /4} \right) \times {\text{D}}2 \times {\text{VTI}} \times {\text{HR}}} \right)/1000$$

The percent difference between the MATLAB and Python average values and standard deviations were then calculated for each parameter. The percent difference was a useful statistic to uniformly evaluate the change in values between MATLAB and Python program analysis as opposed to the numerical change which varied based on the maximum velocity of each individual video’s scale. An f-test was performed to compare the peak velocity values of the two data sets and to determine if the variances of the sets were equal. Finally, a t-test (assuming equal or unequal variance based on the results of the f-test) with a significance level of *p* < 0.05 was then performed to compare the average peak velocity values and CBF. The calculated data was then categorized into groups based on the obstacles present in the video for comparison and evaluation of the improved program’s effectiveness.

### Availability of data and materials

The software described in this study may be downloaded anonymously for non-commercial use from the following repository: https://zenodo.org/record/6308961#.Yh0Uk99OlE4.

## Results

Overall, standard deviation decreased from the MATLAB program to the Python program (Table 1). Standard deviation for peak velocity values decreased by an average of 50.0% for baseline flow videos and 32.1% for hyperemic flow videos, and VTI standard deviation decreased by 51.2% and 35.2% for baseline and hyperemic videos respectively. In individual cases where standard deviation noticeably increased for these parameters, factors such as interfering noise (videos labeled as ‘Top noise’) or incorrect identification of fainter peaks (videos labeled as ‘Missing fainter peaks’) had influenced the calculated standard deviation for the MATLAB program’s output.

**Table 1:** Coronary blood flow pattern variables assessed by the original MATLAB and the new Python programs at baseline and hyperemia.

	Baseline					Hyperemia				
	Average MATLAB values	Average python values	Average % difference	+/− SD	Average % SD difference	Average MATLAB values	Average python values	Average % difference	+/− SD	Average % SD difference
Systolic rise time (ms)	75.58	71.57	0.79	44.55	− 54.61	73.94	62.90	− 10.64	39.60	− 59.22
Diastolic rise time (ms)	23.03	26.73	16.28	36.27	− 11.65	29.05	29.05	1.25	27.80	− 31.23
Diastolic decay time 1 (ms)	34.16	41.94	23.63	26.42	− 11.43	27.91	31.80	9.36	31.88	− 22.92
Diastolic decay time 2 (ms)	61.73	35.30	− 50.94	35.94	− 51.84	64.90	42.16	− 40.48	43.29	− 50.21
Systolic slope (mm/s^2^)	1042.46	443.38	25.82	173.15	− 22.96	2883.07	3686.91	27.85	38.42	7.54
Diastolic slope (mm/s^2^)	24,777.52	10,698.94	− 24.04	68.88	− 29.16	29,405.50	22,284.09	− 11.37	45.06	− 40.01
Decay slope 1 (mm/s^2^)	− 11,183.36	− 3159.46	− 50.62	76.42	− 72.15	− 12,850.25	− 9942.69	− 13.88	49.86	− 68.12
Decay slope 2 (mm/s^2^)	− 5509.69	− 6718.63	23.72	44.74	16.78	− 11,247.26	− 15,374.79	26.34	29.13	9.31
Diastolic velocity (mm/s)	70.99	71.63	179.36	659.25	2.81	211.53	290.45	31.14	34.73	− 15.34
**Peak velocity (mm/s)**	**374.33**	**287.91**	− **6.23**	**51.86**	− **50.00**	**798.03**	**847.01**	**5.50**	**12.37**	− **32.10**
Decay velocity (mm/s)	283.97	187.37	− 13.85	56.69	− 40.78	573.62	561.72	0.09	25.38	− 6.26
Heart rate (BPM)	323.48	359.14	9.31	17.90	− 30.09	320.89	375.22	15.10	16.25	− 33.69
**VTI (mm)**	**24.14**	**22.11**	**4.15**	**49.55**	− **51.19**	**59.04**	**62.24**	**2.99**	**26.41**	− **35.20**

	Baseline—no top noise				Hyperemia—no top noise					
	Average MATLAB Values	Average python Values	Average % difference	+/− SD	Average MATLAB Values	Average python values	Average % difference	+/− SD		
Systolic rise time (ms)	86.71	76.69	− 13.14	20.25	83.04	65.92	− 22.48	20.55		
Diastolic rise time (ms)	25.25	26.74	4.46	22.87	30.63	29.11	− 5.94	21.97		
Diastolic decay time 1 (ms)	33.90	41.48	23.75	27.84	28.85	31.47	7.11	28.77		
Diastolic decay time 2 (ms)	49.65	35.46	− 38.11	28.23	58.08	41.66	− 32.67	27.80		
Systolic slope (mm/s^2^)	201.98	431.48	92.87	129.35	2540.62	3700.73	38.94	23.50		
Diastolic slope (mm/s^2^)	10,238.15	11,007.39	7.71	26.87	21,868.16	23,225.37	7.45	16.99		
Decay slope 1 (mm/s^2^)	− 4179.26	− 3291.31	− 16.58	44.49	− 11,940.33	− 10,032.00	− 14.98	37.36		
Decay slope 2 (mm/s^2^)	− 4166.63	− 6776.93	42.91	26.96	− 11,762.49	− 15,699.49	26.95	18.06		
Diastolic velocity (mm/s)	40.87	73.31	53.85	42.10	222.39	295.15	26.84	17.52		
**Peak velocity (mm/s)**	**234.84**	**287.84**	**19.30**	**13.60**	**773.08**	**863.84**	**10.91**	**8.29**		
Decay velocity (mm/s)	163.14	189.29	13.46	16.22	525.57	576.42	9.90	16.98		
Heart rate (BPM)	324.32	351.83	6.81	19.70	315.76	370.09	15.14	17.26		
**VTI (mm)**	**16.75**	**22.16**	**26.42**	**25.67**	**57.16**	**64.13**	**8.15**	**29.29**		

Relevant values discussed in the text are in [bold].

When examining the two tailed t-tests performed between the peak velocity values of the MATLAB and Python programs, *p* values indicated statistical significance when the Python program made significant improvements to the video’s analysis, such as through removal of top noise, extraction of cycles that were missed in the original analysis, or removal of unrepresentative peaks from the final data set. For cases where the original analysis was accurate, the *p* values suggested that the two data sets were equal. In Table 2, baseline videos which were accurately captured and analyzed by the MATLAB program had an average *p* value of 0.20, which did not indicate significance between the peak velocity values of the two programs. On the other hand, baseline videos that had several cardiac cycles that were not fully captured by the MATLAB program but which were correctly analyzed by the Python program had an average *p* value of 0.004, which did indicate significant differences. Videos with top noise and ECG inaccuracies saw similarly lower *p* values.

**Table 2:** Coronary blood flow peak velocity and VTI as assessed by the original MATLAB and the new Python programs at baseline and hyperemia and under varying circumstances that occur in Doppler videos.

	Baseline		Hyperemia	
	Peak velocity (mm/s)	VTI (mm)	Peak velocity (mm/s)	VTI (mm)
**Accurate analysis**				
Average MATLAB values	282.45	20.75	959.43	62.41
Average python values	293.71	23.42	1000.72	61.89
*p* Value	0.20	0.11	0.26	0.45
Average % difference	4.65	11.74	3.26	− 2.53
+/− SD	7.46	32.23	5.05	7.86
Average % SD difference	− 13.05	− 11.64	− 5.66	− 21.12
**ECG inaccuracies**				
Average MATLAB values	187.64	16.22	653.11	211.50
Average python values	228.54	19.95	717.62	50.44
*p* Value	0.001	0.10	0.15	0.20
Average % difference	20.07	20.80	8.91	− 12.55
+/− SD	9.52	22.72	4.90	10.30
Average % SD difference	− 25.83	− 43.60	− 37.55	− 58.36
**Fainter peaks/unrepresentative cycles**				
Average MATLAB values	251.33	16.01	718.19	49.50
Average python values	342.65	24.68	886.41	79.59
*p* Value	0.004	0.08	**0.01**	0.01
Average % difference	29.63	40.78	20.55	42.79
+/− SD	7.60	22.58	4.25	29.71
Average % SD difference	− 41.24	− 16.06	− 71.06	− 27.76
**Top noise**				
Average MATLAB values	862.55	49.99	829.05	63.03
Average python values	288.15	21.94	757.18	55.79
*p* Value	0.01	0.02	0.44	0.28
Average % difference	− 95.55	− 73.80	− 8.54	− 10.41
+/− SD	27.53	25.33	10.16	9.00
Average % SD difference	− 101.29	− 141.09	− 5.30	− 23.74

**Figure 3:**
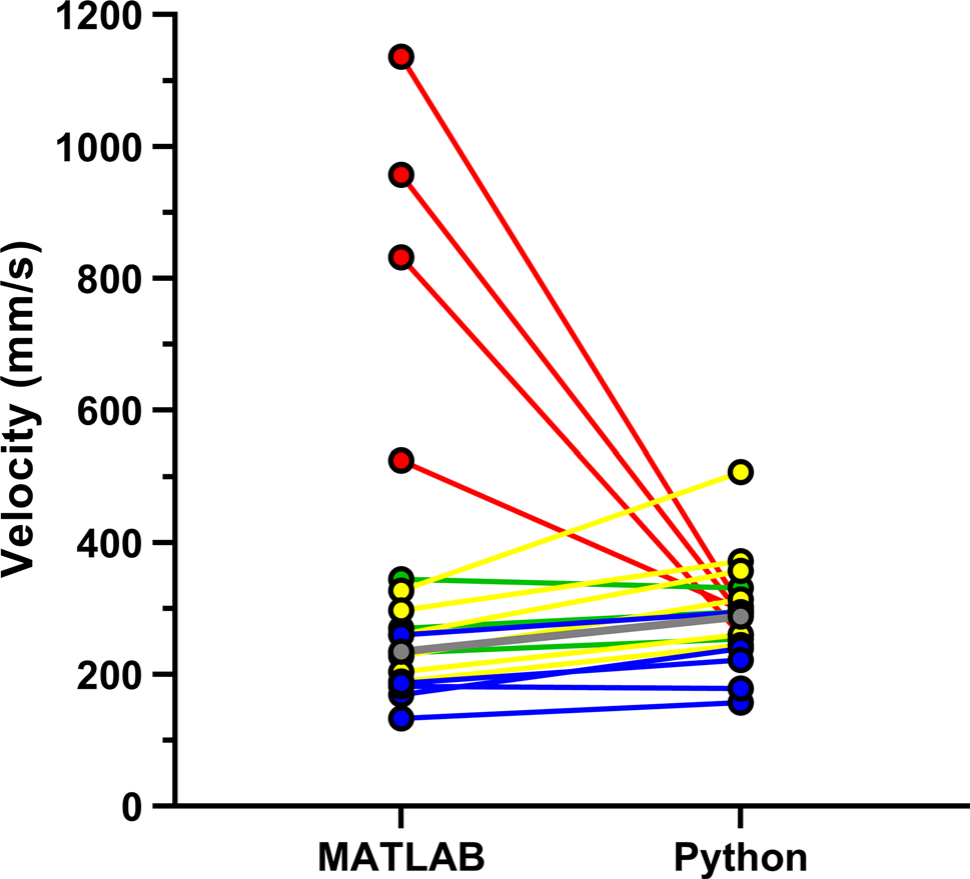
Figure showing the change in average peak velocity values from MATLAB to Python program analysis of each baseline Doppler video. Videos affected by top noise are indicated with red points, accurate analysis is indicated with green points, videos with fainter peaks indicated with yellow, and inaccurate ECG peak identification shown with blue points. The average change in values excluding those top noise videos is represented by the gray line.

Average peak velocity and VTI values tended to increase when using the new algorithm, with the exception of videos where noise at the top of the Doppler region had been captured by the MATLAB program. The change in each individual baseline video’s peak velocity from the MATLAB to the Python program is displayed in Fig. 3, with videos affected by top noise indicated with red dots, accurate analysis indicated with green points, videos with fainter peaks indicated with yellow, and inaccurate ECG peak identification shown with blue points. The overall average peak velocity values, excluding top noise videos, are shown by the gray line. In this figure, the peak velocity for accurately analyzed videos remained similar from the MATLAB to the Python program, while videos with top noise had a significant decrease in peak velocity values and videos with fainter peaks that were not fully captured by the original program tended to have an increase in peak velocity values when analyzed by the updated program. Overall, when not considering top noise videos, peak velocity values increased by an average of 19.3% ± 13.6% and 10.9% ± 8.3% for baseline and hyperemic videos respectively and VTI values increased by 26.4% ± 25.7% and 8.1% ± 29.3% (Table 1).

Several examples of the specific changes that contributed to overall performance improvement are investigated in the rest of this section. Removal of interfering top noise from the Doppler envelope made it possible for the improved program to capture the correct cardiac velocities. The program also added checks to verify ECG peak values so that cardiac cycles weren’t skipped or broken into multiple sections. Finally, the program fully captured fainter cardiac cycles that had been previously overlooked and removed unrepresentative cycles from consideration, both of which were changes that decreased standard deviation and increased average peak velocity and VTI values. When making comparisons, peak velocity and VTI values were selected as the parameters for analysis because they are most representative of the analyzed Doppler region and are the values most commonly utilized in clinical practice.

### Removal of top noise

**Figure 4:**
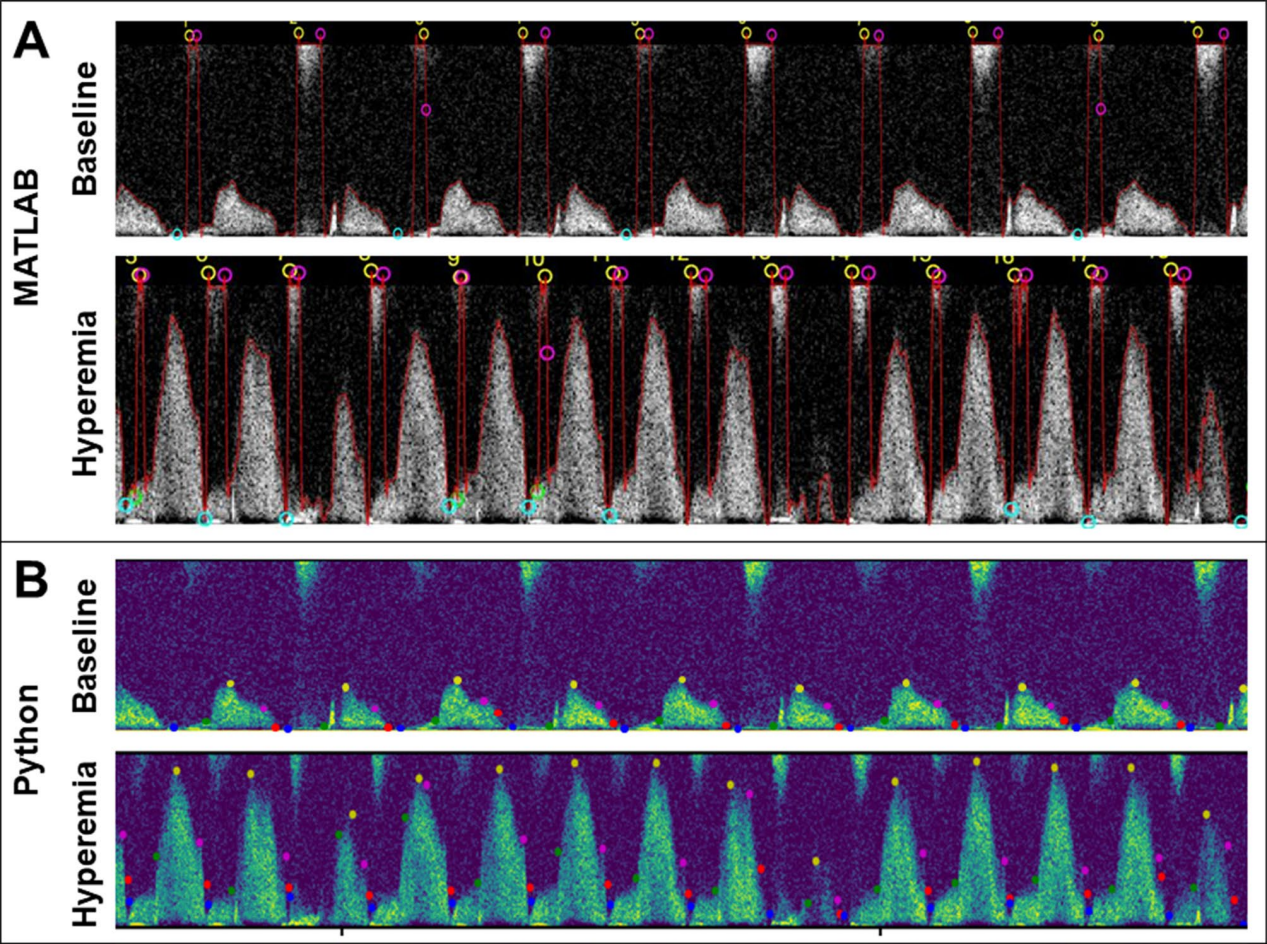
Images displaying the removal of top noise from analysis in the updated Python program. Panel (**A**) displays analysis of representative baseline (above) and hyperemic (below) videos where top noise was included in the Doppler envelope. Panel (**B**) shows the same videos processed by the Python program, where top noise has been discarded from the analyzed pattern. In these images, green points indicate the beginning of the diastolic phase, yellow indicates peak velocity, pink indicates decay velocity, red indicates peak diastolic deceleration, and blue points indicate the end of the cycle.

Many of the videos analyzed in this data set demonstrated the Python program’s ability to identify and remove top noise from the binarized image. To accomplish this, the new algorithm added steps to eliminate any large areas of noise which weren’t close to the baseline of the image. For example, the representative baseline and hyperemic videos displayed in Fig. 4A contained significant top noise which was captured by the MATLAB program. However, when analyzed by the Python program, this noise was removed from consideration in the binarized image and the program could extract accurate values, as shown by the critical points in Fig. 4B.

Removing top noise to more accurately capture the correct velocity values often produced a decrease in average values for the Python program’s values as velocities were no longer forced to the top of the video. On average, baseline videos with top noise had a 95.6% ± 27.5% decrease in average peak velocity values, and hyperemic videos had a 10.4% ± 9.0% decrease in average VTI values (Table 2). This change also contributed to an overall decrease in standard deviation values; standard deviation of peak velocity values decreased by 101.3% and 5.3% for baseline and hyperemic videos respectively. For some individual examples of videos with top noise, standard deviation for peak velocity values increased by up to 88.7% because when processed by the original program, most or all peak velocities were driven up to the same maximum value. However, contrary to indications of increased variability, removal of top noise is a step that allows the new program to more appropriately extract data values from videos which could not be optimally analyzed by the MATLAB program.

### Division of ECG region

**Figure 5:**
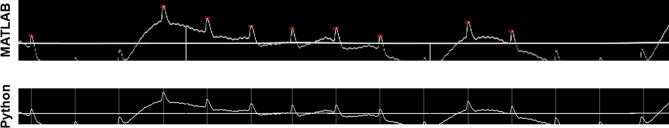
Example of corrected QRS-complex peak identification in the ECG region where the original analysis skipped several peaks. The MATLAB program (above) identifies 9 peaks, indicated by red circles, while the Python program (below) identifies all 15 peaks, indicated by white vertical bars.

**Figure 6:**
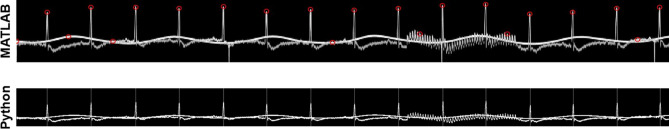
Example of corrected QRS-complex peak identification in the ECG region where the original analysis added several incorrect peaks. The MATLAB program (above) identifies 7 additional peaks, indicated by red circles, while the Python program (below) identifies only the correct 15 peaks, indicated by white vertical bars.

As noted above, before extracting data values, the Doppler region is broken into distinct cardiac cycles by identifying peaks in the ECG region. In cases of unusual ECG readings however, the MATLAB program was unable to identify the correct number of peaks in this region. This resulted in the program missing several QRS complex peaks and thus leaving some cardiac cycles unanalyzed—as depicted in Fig. 5—or in the program selecting multiple peaks within one cardiac cycle, as shown in Fig. 6. In Fig. 5, 9 of the total 15 ECG peaks were identified, while in Fig. 6 an additional 6 peaks were identified along with the 15 correct ones. With added verification and corrections in the Python program, all 15 ECG peaks are identified in Fig. 6, and only the correct 15 peaks are identified in Fig. 6 with no additional peaks. Adding more of the correct ECG peaks to the analysis of this first example decreased standard deviation for peak velocity values by 11.9% and increased the average value by 6.4%, and removing incorrectly added peaks from the second representative example decreased standard deviation of peak velocity values by 31.2% and increased the average value by 34%.

### Identification of fainter peaks

**Figure 7:**
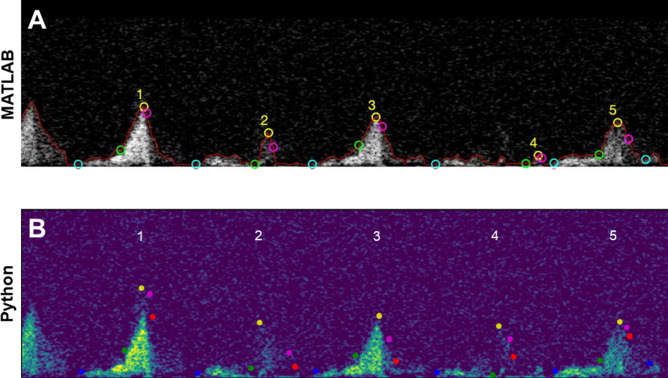
Figures displaying partially and completely captured fainter cycles in the Doppler region. Panel (**A**) above shows cycles 2 and 4 are not fully captured by the MATLAB program, but they are captured and analyzed by the Python program in Panel (**B**) below. As in previous images, green points indicate the beginning of the diastolic phase, yellow indicates peak velocity, pink indicates decay velocity, red indicates peak diastolic deceleration, and blue points indicate the end of the cycle.

The new program was able to overcome some of the difficulty of identifying fainter peaks in the Doppler region, especially in cases where other cardiac cycles were significantly brighter or there was surrounding noise. By employing a more aggressive method of noise removal in the region above the Doppler flow, the Python program was able to accept a lower threshold for binarization in order to capture these fainter peaks without also including surrounding noise in the final binarized image. The original algorithm does not fully capture cycles 2 and 4 when analyzing a representative baseline video, as shown in Fig. 7A, but these peaks are fully captured and analyzed by the Python program in Fig. 7B. This adjustment was incorporated into the program without compromising its ability to exclude unrepresentative flow cycles that result from the coronary artery moving in and out of view of the flow probe.

Correct identification and analysis of these previously overlooked cycles resulted in increased average peak velocity, VTI, and decreased standard deviation. On average, baseline videos in this category increased peak velocity and VTI values by 29.6% ± 7.6% and 40.8% ± 22.6% respectively when incorporating the Python program’s corrections, while standard deviation fell by 41.2% and 16.1% respectively (Table 2).

### Removal of unrepresentative cycles

**Figure 8:**
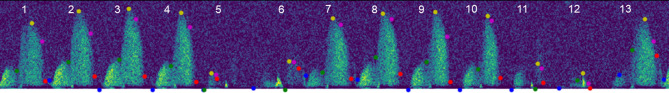
Image generated from the Python program showing unrepresentative cycles in the Doppler region. Cycles 5, 6, 11, and 12 are significantly lower than the surrounding peaks and comparison of these peak values to the average peak velocity leads to rejection from the final dataset after analysis is complete.

The final major improvement made by the Python program to increase accuracy was to remove any unrepresentative cycles from the program’s final output. Unrepresentative cycles were identified by comparing a cycle’s peak velocity and VTI values to the data set’s averages, and if these values were comparatively too low (due to the coronary transiently falling out of the view of the ultrasound during the cardiac cycle) they were removed from the final data set. Removing this information helped to produce more uniform results by not taking into consideration either incorrectly analyzed cardiac cycles or cycles that may have been correctly captured but were not representative of the Doppler region’s overall trends. For example, in Fig. 8, the Python program had correctly identified the Doppler region and analyzed each cardiac cycle, but cycles 5, 6, 11, and 12 were not representative of the rest of the data set, so they were removed from the final data table and subsequent calculation of average values and standard deviation.

Removing the unrepresentative cardiac cycles from this representative video resulted in a 102.21% decrease in standard deviation for the peak velocity and a 57.53% decrease in standard deviation for the VTI. The average peak velocity then increased by 16.8%, and VTI increased by 17.05%. For this analysis, videos in which unrepresentative cycles were removed were grouped with videos which improved identification of fainter peaks because both modifications accomplished the common purpose of removing inaccurate lower values from analysis. Because of this, both adaptations saw a similar increase in peak velocity and VTI values and decrease in standard deviation values.

### Color mode analysis

Additional color mode baseline and hyperemic files analyzed with each set of Doppler videos produced vessel diameters which could be used to calculate blood flow through the measured vessel. Baseline videos identified an average of 19 video frames containing vessels for analysis, and hyperemic videos averaged 51 frames analyzed for vessel diameters. Vessel diameters increased by an average of 34.37% from baseline to hyperemic conditions, which correlates with the increased stimulation of blood flow in the vessel. The new color mode analysis algorithm also generated an image for each video frame containing a measured vessel, and these images could then be inspected for accurate identification.

With the inclusion of vessel diameter calculations, coronary blood flow could also be calculated (Table 3). In agreement with our previous demonstrations by manual analyses^10,11^, the new program was able to resolve significant reductions in CBF in T2DM db/db mice at 12, 16, and 36 weeks of age. Importantly, coronary flow reserve (CFR) was also impaired in db/db mice at 16 and 36 weeks of age compared to normal.

**Table 3:** Coronary blood flow calculated by the new Python program in both normal and T2DM db/db mice at different ages.

Age	Control Db/db			T2DM db/db		
	Baseline (mL/min)	Hyperemia (mL/min)	CFR (H/B)	Baseline (mL/min)	Hyperemia (mL/min)	CFR (H/B)
12 Weeks	1.34 ± 0.22	9.69 ± 1.17	7.57 ± 0.84	0.84 ± 0.11*	5.16 ± 0.94**	6.14 ± 0.92
16 Weeks	1.41 ± 0.14	10.59 ± 0.81	7.61 ± 0.37	0.72 ± 0.09**	4.26 ± 0.26***	6.23 ± 0.54*
36 Weeks	1.04 ± 0.15	6.29 ± 0.58	6.53 ± 0.76	1.30 ± 0.16	4.25 ± 0.60*	3.61 ± 0.65**

Data are mean ± SEM; n = 6 per group; *p < 0.05, **p < 0.01, and ***p < 0.001 versus respective Control.

## Discussion

The early identification of CMD has the potential to allow for the early identification and potential prevention of more serious heart problems such as myocardial ischemia, atherosclerosis, and heart failure. TTDE is an effective and non-invasive method used to assess coronary flow by observing coronary flow patterns, and automatic analysis of coronary blood flow was demonstrated in a previous study by this laboratory to reduce the time required for analysis and the bias typical of manually-analyzed TTDE files^9^. Here, we present improvements to the original program. This improved program took advantage of OpenCV and other Python libraries, and with improved heuristics was able to handle a larger scope of data inputs and accurately analyze more challenging Doppler videos.

This study aimed to refactor the original program, transition to a Python environment for use of the OpenCV and Tensorflow libraries, and to add additional checks and improvements devised from use of the original program in order to increase analysis accuracy and more effectively handle difficult cases. The new code improves handling of interference from top noise, validates identification of ECG peaks, correctly estimates parameters from fainter peaks, and rejects unrepresentative data. In addition, the program functionality was expanded by accepting videos of any pixel height and width and allowing multiple baseline and hyperemic videos to be analyzed in one run.

One major advantage of moving the program to Python was the use of OpenCV for video processing and image analysis. The MATLAB program interpreted each video frame as a cell array of pixel values and analyzed the images to identify the horizontal baseline position, regions of interest to crop to, and threshold values for binarization. The Python program utilized functions of the OpenCV library to accomplish these steps, as well as for grayscale conversion and for applying a gaussian filter and dilation to the Doppler region before calculating the binarization threshold. The findContours function was especially useful in adding modified heuristics to identify top noise and other noise objects that needed to be removed.

The Python program took advantage of several other Python libraries for specific analysis steps; numpy was used for array manipulation and mathematical calculations as parsed images were treated as arrays of pixel values, tkinter was used to create GUIs for user interaction, and matplotlib was used to plot critical values on the images of the coronary flow pattern that were saved from each processed video. As future developments are added, the Python environment will be able to utilize TensorFlow, scikit-learn, and other libraries for further data analysis and machine learning algorithms.

The data and examples provided specifically demonstrate the program’s ability to remove top noise, to improve identification of peak ECG values, to better capture fainter cardiac cycles, and to remove unrepresentative cardiac cycles from analysis. Overall, this resulted in decreased standard deviations from the original to the improved program. This decrease in standard deviation indicates a more uniform analysis of each cardiac cycle and the proper removal of inaccurate cycles. Increased average peak velocities and VTI values in cases except those dealing with top noise interference also demonstrate the program’s improved analysis as unrepresentative cycles were removed and fainter peaks that had previously been only partially captured were fully analyzed.

Performing open software development strengthens the research community. Any research group can either contribute to this project to improve the software or they are free to develop a different tool using our work as a foundation. Open software due to its transparency also increases reproducibility in research. The software can be directly examined without any delay should a specific need to know arise when assessing research that uses the software. As both our software and Python are free, we believe this also adds to the portability and potential impact of our work.

### Related studies

A handful of similar programs have been developed to use automated analysis to reduce processing time and parameter variability. Many of these programs rely on partial-automation combined with expert analysis to enhance accuracy without removing manual intervention. For example, a program developed in MATLAB was used to crop video frames to the region of interest containing the Doppler envelope and apply a binarization threshold adjusted by the user, similar to the verification included in this current study’s algorithm^12^. The program analyzed the Doppler region in frames containing three heartbeats at a time, with each frame taking between 10 and 40 s to analyze, and calculated a subset of the parameters found in in this study; peak diastolic velocity, peak diastolic acceleration, beginning diastolic phase, peak systolic velocity, and peak diastolic deceleration. When analyzing 200 videos from 100 patients, linear regression indicated strong correlation to manual analysis in PSV (*r* = 0.986, *p* < 0.0001, SE = 2.51 cm/s) and PDV (*r* = 0.998, *p* < 0.0001, SE = 1.58 cm/s).

A similar study focused on removing all manual intervention from Doppler aortic flow analysis in order to minimize bias and analysis time^13^. The program tested Doppler strips of several heartbeats at a time and followed a similar procedure of cropping to the region of interest, binarizing the image to capture the Doppler envelope, and extracting critical values from each cardiac cycle. The program was advantageous in that it also didn’t rely on QRS complex peaks in the ECG region to divide the Doppler region into cardiac cycles, but instead relied only on the Doppler data to separate cycles. However, the program did not account for some of the added heuristics implemented in this current study, such as discarding unrepresentative peaks from consideration and removing top noise from the Doppler envelope. Due to these challenges, when analyzing heartbeats from 18 patients through 9 manual and 1 automatic analysis, the automated measurements were outside the range of manual values 9.5% of the time for VTI values and 3.9% of the time for peak velocity values. However, overall this program displayed strong correspondence in identified VTI and PV values to expert analysis, and saw a tenfold reduction in time for analysis, as opposed to 30-fold reduction seen by the programs in this current study.

### Limitations

Removing user interaction in favor of more computer automation would help to increase consistency, especially in identification of the correct threshold level for image binarization. However balancing user interactivity with complete automation is necessary for evaluators to adjust for errors and special cases, so allowing for a manual adjustment of the threshold value for image binarization is most effective. The option to adjust the binarization threshold is a critical element that needs to remain in order for a trained expert in coronary flow to assess the suitability of the pattern moving in and out of view of the Doppler during the cardiac cycle—a phenomenon that’s difficult to automate.

## Conclusions

Comparison of the data values and plots generated from the original MATLAB and improved Python programs serve to demonstrate the increased accuracy of the updated algorithm to automatically measure CBF, specifically its ability to process a wider range of video sizes, special cases, and inaccurate readings that the original program did not have checks to handle. The improved program is able to remove top noise and other large noise artifacts, to verify the correct identification of ECG peaks, to better capture fainter peaks in the Doppler region, and to remove unrepresentative values from the final set of parameters. The program accepts any video pixel height and width and allows for the analysis of more than one baseline and hyperemic video at a time. Videos that had already been accurately analyzed by the MATLAB program continued to output similar data values, while videos that were corrected showed decreased standard deviation and increased peak velocity and VTI values. Finally, the improved program was able to automatically resolve differences in CBF in a mouse model in which we’ve previously demonstrated impaired CBF. The program has achieved its goal of improving algorithm heuristics in order to better handle special cases, and can be used by examiners as an efficient, fast, and exact way to automatically analyze coronary Doppler echocardiograms.
